# Different Effects of the Immunomodulatory Drug GMDP Immobilized onto Aminopropyl Modified and Unmodified Mesoporous Silica Nanoparticles upon Peritoneal Macrophages of Women with Endometriosis

**DOI:** 10.1155/2013/924362

**Published:** 2013-12-24

**Authors:** Yuliya Antsiferova, Nataliya Sotnikova, Elena Parfenyuk

**Affiliations:** ^1^Federal State Institution “Ivanovo Research Institute of Maternity and Childhood named V.N.Gorodkov” of Healthy Ministry of Russian Federation, Pobedy Street 20, Ivanovo 153045, Russia; ^2^G.A. Krestov Institute of Solution Chemistry of Russian Academy of Sciences, Akademicheskaya 1, Ivanovo 153045, Russia

## Abstract

The aim of the present work was to compare in vitro the possibility of application of unmodified silica nanoparticles (UMNPs) and modified by aminopropyl groups silica nanoparticles (AMNPs) for topical delivery of immunomodulatory drug GMDP to the peritoneal macrophages of women with endometriosis. The absence of cytotoxic effect and high cellular uptake was demonstrated for both types of silica nanoparticles. The immobilization of GMDP on the UMNPs led to the suppression of the stimulatory effect of GMDP on the membrane expression of scavenger receptors SR-AI and SR-B, mRNAs expression of NOD2 and RAGE, and synthesis of proteolytic enzyme MMP-9 and its inhibitor TIMP-1. GMDP, immobilized onto AMNPs, enhanced the initially reduced membrane expression of SRs and increased NOD2, RAGE, and MMP-9 mRNAs expression by macrophages. Simultaneously high level of mRNAs expression of factors, preventing undesirable hyperactivation of peritoneal macrophages (SOCS1 and TIMP-1), was observed in macrophages incubated in the presence of GMDP, immobilized onto AMNPs. The effect of AMNPs immobilized GMDP in some cases exceeded the effect of free GMDP. Thus, among the studied types of silica nanoparticles, AMNPs are the most suitable nanoparticles for topical delivery of GMDP to the peritoneal macrophages.

## 1. Introduction

Nowadays the immunomodulators are widely used for the treatment of different diseases with proved immune etiology. It was demonstrated that immune mechanisms are directly involved in the endometriosis pathogenesis [1]. Endometriosis affects ~10% of women of reproductive age and often results in infertility [2]. It was shown that the development and growth of endometriotic lesions in the peritoneal cavity are associated with the impaired function of peritoneal macrophages [3, 4]. Macrophages of women with endometriosis are incapable effectively to recognize and eliminate the viable endometrial cells from peritoneal cavity [3]. Molecular mechanisms of this phenomenon are not elucidated yet. Likely, the impairment of the expression of macrophages specific membrane receptors might be responsible for ineffective removing of endometrial cells from peritoneal cavity by macrophages. In our previous work we have shown that expression of the membrane scavenger receptors SR-A1 and SR-B, responsible for the removing of cellular debris from peritoneal cavity, by peritoneal macrophages of women with endometriosis is significantly reduced in comparison to that in healthy fertile women [5]. Experimentally we had also demonstrated that the decreased expression of these scavenger receptors on the surface membrane of macrophages resulted in the impairment of macrophages interaction with the autologous endometrial cells [5]. It is known that scavenger receptors belong to the large family of signaling pattern-recognition receptors or PRRs [6]. It is well documented now that activation of phagocyte cells, which are considered as the major effectors cells of host defense system, depends on the ability of phagocytes to recognize different groups of exogenous and endogenous antigens using the special PRRs [6]. So, it can be proposed that the appropriate correction of PRRs expression by peritoneal macrophages might improve their interaction with endometrial cellular debris in peritoneal cavity of women with endometriosis and significantly increase the efficacy of medical treatment of endometriosis.

Despite the intensive work in the field of new immunomodulatory drugs development, the list of clinically approved immunomodulators is still rather short. Muramyl dipeptide (MDP), the minimal active fragment of peptidoglycan of the cell wall of Gram-positive and Gram-negative bacteria, has gained much attention in the last years due to its significant immunomodulatory effect upon phagocytes [7]. It was shown that the phagocyte's response to MDP is mediated via one of the PRRs, nucleotide-binding oligomerization domain 2 (NOD2) [8]. But the precise mechanisms of MDP action are still unknown. A series of derivatives of MDP have been designed and synthesized. One of these derivatives is glucosaminyl muramyldipeptide (N-acetylglucosaminyl-N-acetylmuramyl-L-alanyl-D-isoglutamine) or GMDP [9]. Now GMDP is used as a drug with immunomodulatory action. It has been shown that GMDP strongly stimulates reactions of adaptive and especially innate immune responses [10]. This drug has been widely used for therapy of different chronic infections and autoimmune diseases but never has been used for endometriosis treatment. Taking into account the suggestion that the development of endometriosis is associated with the impaired function of the peritoneal macrophages, we proposed that GMPD can positively influence the initially reduced expression of scavenger receptors by peritoneal macrophages of women with endometriosis. We also suggested that GMDP might act via other macrophages PRRs and its inhibitors. However, GMDP has low bioavailability (7%–13%). It can be proposed that the drug effect may be intensified by immobilization of GMDP onto nanoparticles, which may alter drug's reactivity, strength, and behavior in vivo [11]. Modern advances in nanotechnology can allow delivery of a drug to a targeted tissue, release of a drug at a controlled rate, treatments for drug detoxification [11]. Therefore the other aim of our work was to develop the most suitable nanocarrier for GMDP and to compare the effects of free GMDP and the GMDP, immobilized onto nanoparticles, on functional activity of the peritoneal macrophages of women with endometriosis.

In the literature various materials are proposed as drug carriers [11, 12]. Among them sol-gel silica materials have received much attention because they are biocompatible in vivo as they are readily degradable inside the body [13] and are not subjected to microbial attack. Amorphous colloidal porous silica has been proposed as a suitable drug delivery system due to its attractive properties. In recent years, much attention has been paid to mesoporous silica nanoparticles (with a pore diameter of 2–50 nm) as carriers for controlled drug delivery. Mesoporous silica shows many promising characteristics such as uniform and tunable pore and particle size, exceptionally high surface area, often exceeding 1000 m^2^/g, and large pore volume, stable structure which is resistant to heat, pH, mechanical stress [14, 15]. Therefore in the present work mesoporous silica nanoparticles (UMNPs) were synthesized via sol-gel procedure. Another type of silica nanoparticles that has been studied in the present work is aminopropyl functionalized silica nanoparticles (AMNPs), which were prepared via cocondensation sol-gel process of tetraethoxysilane (TEOS) and 3-aminopropyl triethoxysilane. They are also considered as promising drug carriers [16, 17]. The choice of these types of silica nanoparticles is not random. According to the literature data [18–20], amorphous silica and cationic silica nanoparticles are accepted as having low cytotoxicity and genotoxicity. However, the toxicity of silica nanoparticles is affected by many factors: surface chemistry, porosity, particle size, concentration, time of incubation and mode of administration into human organism [18–21]. A measure of cytotoxicity of each material must be clearly tested because the extent to which un- and functionalized mesoporous silica are toxic to mammalian cells has not yet been fully explored. Therefore we have studied the effect of the UMNPs and AMNPs on the viability and functional activity of the peritoneal macrophages. Various techniques are applied for drug immobilization, including adsorption, covalent attachment, and entrapment in polymers [22]. Drug adsorption on nanoparticles due to noncovalent interactions is easy to perform and widely used for drug loading. It is an attractive way to bind, deliver, and release actual drug without needing any triggers [23].

So, in the present work we attempted to immobilize GMDP on the different types of silica nanoparticles by adsorption from isotonic solution with subsequent estimation of the effects of obtained GMDP/silica nanocomposites as well as free GMDP upon macrophage expression and synthesis of: (i) several PRRs molecules (scavenger receptors SR-AI (CD204) and SR-B (CD36), NOD2 receptors, RAGE (receptor for advanced glycation end products)), (ii) suppressor of cytokine signaling 1 (SOCS1), which is known as a negative-feedback regulator of PRRs-induced signaling, and (iii) proteolytic enzyme MMP-9 (matrix metalloproteinase-9) and its tissue inhibitor TIMP-1, which are the important enzymes, providing interaction of phagocytes with its targets during phagocytosis process.

## 2. Materials and Methods

### 2.1. Chemicals

Tetraethoxysilane (TEOS) (high purity grade, Russia), 3-aminopropyl triethoxysilane (APTES) (Aldrich, 99%), sucrose (ICN Biomedicals, >99% purity), trypan blue solution (0.1%; Sigma-Aldrich, USA), nitrotetrazolium blue (Sigma-Aldrich, USA), zymosan (Sigma-Aldrich, USA), sheep IgG, labeled by fluorescein isothiocyanate (FITC) (Sorbent, Moscow), FITC-conjugated monoclonal antibodies anti-HLA-DR, CD14, CD11b, CD95, CD36, CD204 (Beckman Coulter), and immunomagnetic beads charged anti-CD14 mouse IgG (Dynabeads CD14, Invitrogen by Life Technologies AS, Oslo, Norway) were used in the work. N-acetylglucosaminyl-N-acetylmuramyl-L-alanyl-D-isoglutamine (GMDP) was kindly given by “Peptek” (Institute of Bioorganic Chemistry of RAS).

Sodium chloride (high purity) and double distilled water were used for preparation of isotonic solution. Potassium bromide (Acros, 99+%, IR grade) was dried at 250°C before use.

Commercial kit “Oncoscreen” (“GenoTechnology”, Moscow, Russia) was used to perform the reaction for total RNA isolation and reverse transcription. Sets of primers, probes, and enzymes solutions for estimation of MMP-9 and TIMP-1 mRNAs expression (Fractal Bio, St.-Petersburg, Russia) and for detection of NOD2, RAGE, and SICS1 mRNAs expression (Sintol, Moscow, Russia) by peritoneal macrophages were used in our study.

### 2.2. Synthesis and Characterization of Silica Materials

The unmodified silica was synthesized via HCl-catalyzed sol-gel procedure of TEOS in the presence of pore forming agent (sucrose) as described elsewhere [24]. But in our procedure of the synthesis the silica precursor was not prehydrolyzed. After removal of the pore forming agent by water extraction and drying the mesoporous, unmodified silica was obtained.

The aminopropyl modified silica was synthesized via hydrolysis and cocondensation of precursor mixture (TEOS: APTES = 3 : 1 v/v) as described earlier [25].

To indentify the synthesized silica materials, the FTIR spectra of the samples were recorded on an Avatar 360 FTIR ESP spectrometer in a range of 4000–400 cm^−1^. The powders were milled and pressed into discs with KBr. The FTIR spectra of the sucrose and fructose containing materials are also recorded.

Small-angle X-ray scattering (SAXS) study is used to measure the periodicity of materials' structure. The synthesized silica powders were investigated by SAXS. The experiments were performed with a diffractometer DRON-2 (Russia) operating at 40 V (Cu-K*α* radiation, *λ* = 0.154 nm). The spectra were recorded in the 2*θ* range of 8 to 57° with 2*θ* step size of 1°.

In order to study effects of the silica nanoparticles on functional state of peritoneal macrophages, the suspensions of the silica nanoparticles with an average radius of 50 nm were prepared in isotonic solution as described earlier [25]. The size and concentration of the particles were determined from turbidity (*τ*) measurements. The turbidity spectra of the suspensions were recorded with a spectrophotometer Agilent 8453 in the range of 400–600 nm using 10 mm quartz cuvettes. Theory of the method is presented in [26, 27]. In brief, optical densities (A) of the suspension were measured as function of wavelength (*λ*) and plotted in the double-logarithmic coordinates (ln⁡*D* − ln⁡*λ*) to determine *n* as a slope of the plot. *n* is a complex function of the particle size and their relative refractive index (*m* = *n*_sil_/*n*_*m*_, *n*_sil_, and *n*_*m*_ are the refractive indexes of the silica particles and surrounding medium, resp.), *n*(*α* = 2*πr*/*λ*, *m*). The *n*_sil_ value is reported in [26]; the *n*_*m*_ value of isotonic solution was measured and found to be 1.3360. So, the *m* value of the silica particles in isotonic solution is *≈*1.10. Knowing *n*, one can obtain the *α* value according to the data tabulated in [27]. The average radius of the particles was calculated as

(1) $$\begin{array}{c} r=\frac{\alpha \lambda }{2\pi m}. \end{array}$$

The turbidity *τ* is proportional to the number concentration *N* of particles and their scattering efficiency *Q*:

(2) $$\begin{array}{c} \tau =\frac{2.3D}{l}=\pi r^{2}NQ. \end{array}$$

The *Q* values can be calculated according to the data tabulated in [27]. The number concentration of the particles *N* (per m^3^) was calculated from (2).

### 2.3. Immobilization of GMDP on Silica Nanoparticles

The immobilization of GMDP on the silica nanoparticles was carried out by addition of the drug solution (isotonic solution) to the suspensions of the nanocarriers (UMNPs or AMNPs with a radius of 50 nm). The concentrations of the UMNPs and AMNPs in suspensions were found to be 3.8 · 10^−10^ mM and 1.5 · 10^−10^ M, respectively. Molar ratio of the nanoparticles to GMDP was 1 : 10.

### 2.4. Isolation of Peritoneal Mononuclear Cells

Peritoneal fluid of 40 women with endometriosis (32 with mild endometriosis (stage II of disease according to the classification of American Society for Reproductive Medicine) and 8 with severe endometriosis (III-IV stages of disease)) who underwent laparoscopic examination for pelvic pain or infertility was used as the material. Informed consent was given from each woman participating in our study, according to local Ethic Committee protocol. All patients ranged in age from 20 to 40 years and were not taking any hormone therapy at least 3 months prior to collection of the samples. Samples of peritoneal fluid were aspirated into the sterile tubes from the Douglas pouch during laparoscopic surgery and immediately used for the investigation. The standard procedure of isolation of the peritoneal mononuclear cells (MNC) by centrifugation in density gradient of Ficoll-Urografin (d-1,078) was performed. According to flow cytometry data obtained fractions of peritoneal MNC contained macrophages with phenotype CD45+CD14+ (70–80%) and lymphocytes with phenotype CD45+CD14− (20–30%).

### 2.5. Estimation of the Effect of Silica Nanoparticles on Viability and Functional Activity of Macrophages

Peritoneal MNC in concentration of 2 × 10^6^ cells/mL was incubated in RPMI 1640 medium in the presence of the synthesized silica nanoparticles for 24 h at 37°C and 5% CO_2_. According to the literature data, concentration of the nanoparticles in the culture media was 400 *μ*g/10^6^ cells/mL [28]. Cells, which were incubated in RPMI 1640 medium in the absence of the nanoparticles at the same conditions, were used as control. After incubation the viability of macrophages was estimated using the trypan blue dye exclusion assay, and the membrane expression of some functional molecules (HLA-DR, CD11b, CD95, CD36, CD204) was defined using monoclonal antibodies by flow cytometry method. Production of the reactive oxygen species by macrophages after its incubation with the silica nanoparticles was estimated using the test of spontaneous (NBTsp) and zymosan stimulated (NBTst) reduction of nitrotetrazolium blue.

### 2.6. Estimation of the Intensity of Interaction between Peritoneal Macrophages and Silica Nanoparticles

To estimate the level of cells, reacting with the silica nanoparticles, the peritoneal MNCs (100 *μ*L) were incubated with 5 *μ*L of the IgG-immobilized silica particles for 1 and 24 h at 37°C and 5% CO_2_. Immobilization of the labeled IgG onto the silica nanoparticles was carried out from isotonic solution. To suspension of the nanoparticles a solution of the protein was added (2 : 3 v/v). The level of the peritoneal macrophages reacted with the silica nanoparticles was estimated by flow cytometry as the amount of fluorescence-bright cells.

### 2.7. Estimation of the Effect of Free GMDP and GMDP/Silica Nanocomposites on Functional State of Peritoneal Macrophages

The peritoneal MNCs were incubated in vitro in 0,5 mL RPMI 1640 medium either in the presence of free GMDP (2 *μ*g/mL) or GMDP immobilized on the UMNPs and AMNPs (50 *μ*L of solution) for 24 h at 37°C and 5% CO_2_. After incubation, the surface expression of CD36 *и* CD204 molecules by the macrophages was estimated by flow cytometry method. The parameters of cells incubated only in the presence of culture media RPMI 1640 were used as the control.

### 2.8. Flow Cytometry

One hundred thousand cells per tube were used for immunofluorescence staining. Cells were incubated with 5 *μ*L of the FITC-conjugated monoclonal antibodies (mAbs) or with IgG-immobilized silica particles for 30 min at room temperature. After incubation cells were washed by isotonic solution at 10 min and fixed according to standard procedure. Flow cytometry analyses were performed on FACScan (Becton Dickinson, USA) using Cell Quest-Pro software (Becton Dickinson, USA). Data from forward versus side scatter was obtained to analyse the CD14+ macrophages population. The data were presented as the percentage of stained cells in the macrophage population.

### 2.9. Real-Time Reverse-Transcription Polymerase Chain Reaction (RT-PCR)

For RT-PCR assays we used pure CD14+ population of peritoneal macrophages. After incubation of peritoneal MNC in the presence of free GMDP or GMDP, immobilized onto nanoparticles, the positive separation of CD14+ macrophages using immunomagnetic beads, charged anti-CD14 antibodies (Invitrogen by Life Technologies AS, Oslo, Norway), was performed according to manufacturer's protocol. Total RNA was isolated from pure fraction of peritoneal macrophages using the standard acid guanidinium thiocyanate-phenol-chloroform method. RNA was converted to complementary DNA (cDNA) using commercial kit “Oncoscreen” (GenoTechnology, Moscow, Russia). Reverse transcription was performed at 70°C for 3 min and 37°C for 90 min.

For quantitative estimation of the mRNA expression of *β*2-microglobulin (housekeeper gene), MMP-9 (matrix metalloproteinase-9), TIMP1 (tissue inhibitor of matrix metalloproteinase-1), NOD2 (nucleotide-binding oligomerization domain 2), RAGE (receptor for advanced glycation end products), SOCS1 (suppressor of cytokine signaling 1), commercial sets of solutions, primers, and probes were used. For the thermocycle reactions and the detection of the fluorescence signals, iCycler iQ Multi-Color Real Time PCR detection System (BIO-RAD Laboratories, California, USA) was used.

Sequences of corresponding genes cloned were used as controls. For each sample, the amount of copy numbers of *β*2-microglobulin and specific genes was determined from the appropriate standard curve generated by iCycler iQ software. The amount of specific gene was subsequently divided by the *β*2-microglobulin gene amount to obtain normalized specific gene value. Results were presented as the ratio in a sample × 10^3^ per *μ*L for all genes.

### 2.10. Statistics

Results of the immunologic study were presented as the mean ± standard error. Data were analyzed using STATISTICA 6.0 software. All variables were checked for normal distribution with the Kolmogorov-Smirnov test. Student's *t*-test was used to compare results between groups with normal distribution. Mann-Whitney test was used to compare results between groups with nonnormal distribution. The level of significance was set at *P* < 0.05.

## 3. Results and Discussion

### 3.1. Characterization of Synthesized Silica Materials (UMNPs and AMNPs)

**Figure 1:**
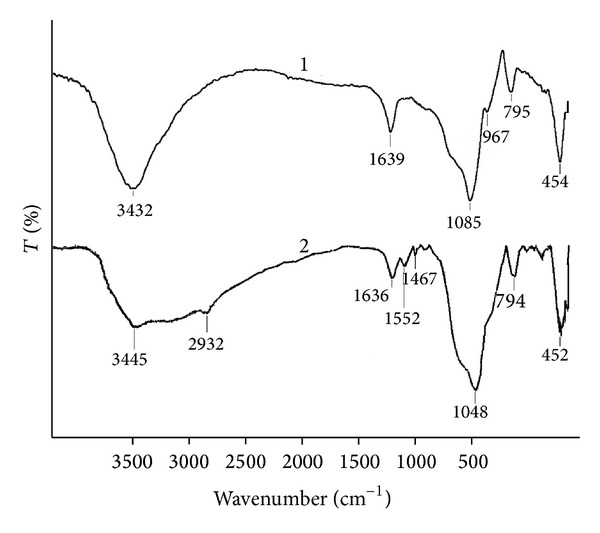
FTIR spectra of unmodified silica (1) and aminopropyl modified silica (2).

Figure 1 shows FTIR spectra of the synthesized silica materials. The characteristic bands assigned to silica are presented in the spectra: the broad peaks at 3432 cm^−1^ and 3445 cm^−1^ are attributed to O–H stretching and adsorbed water [29, 30], the peaks at 1636 cm^−1^ and 1639 cm^−1^ are ascribed to O–H bending of adsorbed water [29, 30], and the strong peaks at 1048 cm^−1^ and 1085 cm^−1^, at about 795 cm^−1^ and 454 cm^−1^ can be assigned to the stretching vibrations of the mesoporous framework (Si–O–Si) [29, 30], and the peak at 960 cm^−1^ is attributed to Si–OH bond stretching [29]. As can be seen from Figure 1, the spectrum of aminopropyl modified silica (spectrum 2) exhibits the band at 1552 cm^−1^ assigned to vibrations of N–H bond of amino groups [30] as well as the bands at 2932 cm^−1^ and 1467 cm^−1^ are assigned to stretching and bending vibrations of aminopropyl CH_2_ groups [31]. Thus, the FTIR spectra indicate that the synthesized silica particles have different surface functional groups.

The materials were studied by small angle X-ray scattering (SAXS) method. The spectra showed a broad peak corresponding to amorphous silica (2*θ* ≈ 27–50°). So, the prepared materials are amorphous. As has been mentioned above, amorphous silica has low toxic effects upon living organisms [18–20].

### 3.2. Investigation of the Influence of Different Silica Nanoparticles on Viability and Functional Activity of Peritoneal Macrophages

According to trypan blue dye exclusion assay data, the viability of macrophages after 24 h incubation with studied silica nanoparticles did not change significantly. It was found to be about 85%–95% after incubation of cells with both UMNPs and AMNPs.

The data presented in Table 1 show that incubation of the peritoneal macrophages in the presence of the UMNPs and AMNPs for 24 h did not significantly influence the expression of functional membrane molecules by peritoneal macrophages.

The amount of HLA-DR+, CD11b+, and CD95+ macrophages after its incubation with the silica nanoparticles of all investigated types was similar to that in the control (*P* > 0.05 in all cases). No significant changes in the expression of scavenger receptors (CD204 and CD36 molecules) and spontaneous and zymozan-stimulated NBT-activity of the macrophages were observed after incubation of the macrophages with the studied silica nanoparticles. So, the synthesized silica nanoparticles are nontoxic in relation to the peritoneal macrophages.

### 3.3. Investigation of the Intensity of an Uptake of Silica Nanoparticles by Peritoneal Macrophages

We estimated the cellular uptake of UMNPs and AMNPs by peritoneal macrophages after its cocultivation for 1 h and 24 h with FITC-labeled silica nanoparticles using flow cytometry. The data characterizing the intensity of interaction between the peritoneal macrophages and studied silica nanoparticles are presented in Table 2.

After incubation of the macrophages with the UMNPs for 1 h, the amount of the immune cells interacting with the nanoparticles was found to be about 82%. After incubation for 24 h, these values were found to be approximately 94% for this type of nanoparticles, and these results were statistically higher than analogous data obtained after 1 h incubation (*P* < 0.05). Thus, the cellular uptake of UMNPs by macrophages is intensive and time dependent.

**Figure 2:**
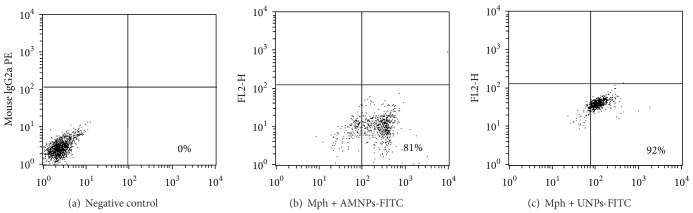
Representative dot plot of macrophages, reacting with FITC-labeled silica nanoparticles after 24 h incubation. (a) Negative control, cells were incubated in the presence of sheep FITC-labeled immunoglobulin, (b) distribution of macrophages according to FITC-negative (low left corner) and FITC-positive cells (low right corner) after 24-hour incubation of macrophages with AMNPs, (c) distribution of macrophages according to FITC-negative (low left corner) and FITC-positive cells (low right corner) after 24-hour incubation of macrophages with UMNPs.

These results have been compared with those for the AMNPs (Table 2). It should be noted that the macrophages interact more intensive with the UMNPs in comparison with the AMNPs (Figure 2). As the nanoparticles of these two types have the same size but different surface properties, it is likely that the last factor plays an important role.

### 3.4. Studies of Effects of Free GMDP and the GMDP Immobilized on UMNPs and AMNPs on the Functional State of the Peritoneal Macrophages of Women with Endometriosis

**Figure 3:**
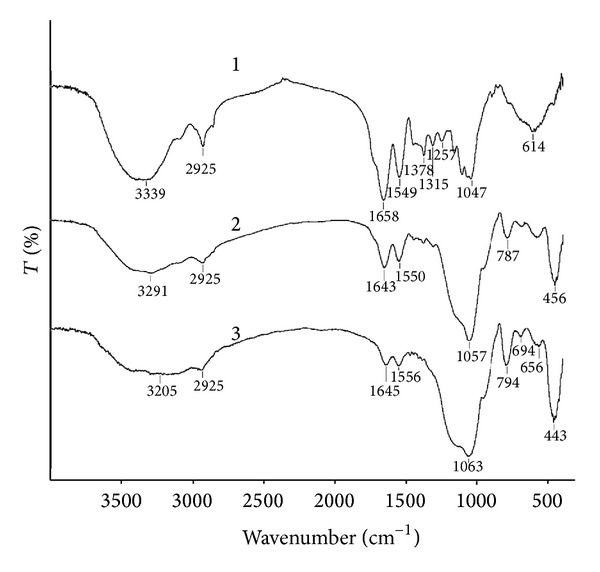
FTIR spectra of free GMDP (1), GMDP immobilized on unmodified silica (2), GMDP immobilized on aminopropyl modified silica (3).

At the next step of our work we immobilized the GMDP on unmodified mesoporous silica nanoparticles (UMNPs) and the aminopropyl modified silica nanoparticles (AMNPs) as nanocarriers. The formation of the composites of GMDP with the silica nanoparticles is confirmed by FTIR spectroscopy.  Figure 3 demonstrates the FTIR spectra of free GMDP and GMDP immobilized onto the silica materials.

The bands at 1658 cm^−1^ and 1549 cm^−1^ are the characteristic for GMDP (spectrum 1). They are assigned to amide I band (mainly C=O stretch) and amide II band (C–N stretch coupled with N–H bending mode) [32, 33]. As can be seen from the spectrum 2, the immobilization of GMDP on the UMNPs is confirmed by the appearance of the bands at 2925 cm^−1^, 1643 cm^−1^, and 1550 cm^−1^ assigned to C–H stretching mode [31], amide I and II bands, respectively. It should be noted that at the immobilization amide I band is shifted (Δ = 15 cm^−1^) towards low-frequency region whereas no change was observed in amide II band. The immobilization of the drug on the AMNPs (spectrum 3) is accompanied by a shift of amide I band (Δ = 13 cm^−1^) towards low-frequency region and a shift of amide II band (Δ = 7 cm^−1^) to high-frequency region. The shift of amide I band indicates on participation of C=O group in the interactions between the drug and the particles. However the interpretation of the shift of amide II band is doubtful because of overlapping of this band and the N–H deformation band of the aminopropyl groups in the region. The shifts testify about adsorption of GMDP on the silica materials via hydrogen bonding [33]. So, the FTIR spectra indicate on successful immobilization of GMDP on the UMNPs and AMNPs.

The effects of free GMDP and the GMDP, immobilized on UMNPs and AMNPs on the membrane expression of scavenger receptors SR-AI (CD204) and SR-B (CD36) by peritoneal macrophages of women with endometriosis, were studied. The obtained results are presented in Table 3.

**Figure 4:**
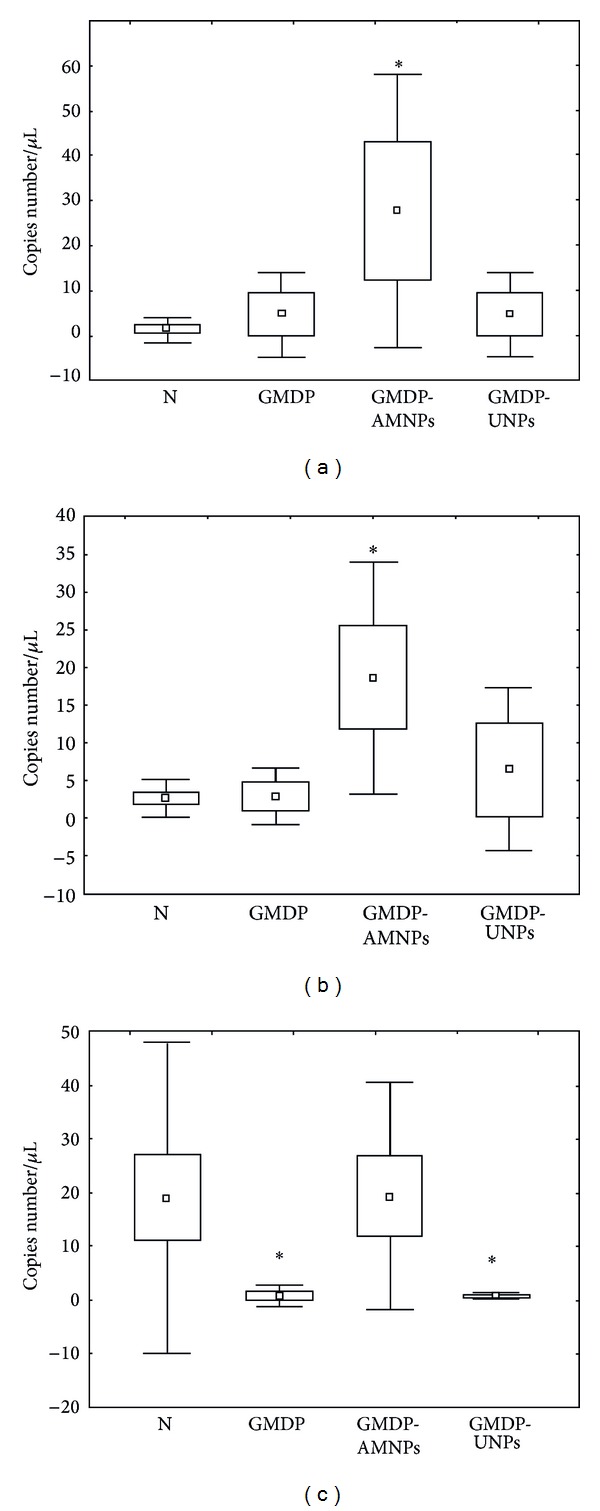
The influence of GMPD, immobilized upon the different silica nanoparticles, and free GMPD on the NOD2, RAGE, and SOCS1 mRNAs expression by peritoneal macrophages of women with endometriosis. (a) The influence of GMPD on NOD2 mRNA expression. (b) The influence of GMDP on RAGE mRNA expression. (c) The influence of GMPD on SOCS1 mRNA expression. (Notes: C—control (incubation in RPMI 1640 medium only), GMDP—free form of GMDP, AMNPs-GMDP, immobilized onto the AMNPs, UMNPs-GMDP, immobilized onto the UMNPs, results are presented as the mean ± standard error; *differences in comparison to the control values are statistically significant, *P* < 0.05).

As we can be seen from Table 3, after 24-hour incubation of the peritoneal macrophages of women with endometriosis in the presence of free GMDP, the amount of the macrophages with surface expression of CD36 and CD204 molecules was significantly increased in comparison to that in the control (*P* < 0.01, *P* < 0.001, correspondently). Thus, GMDP stimulates the expression of the scavenger receptors by the peritoneal macrophages of women with endometriosis. The incubation of the macrophages with GMDP immobilized on AMNPs also results in increasing the expression of CD36 and CD204 molecules by the peritoneal macrophages in comparison to the control values, and in both cases the differences were statistically significant (*P* < 0.001, *P* < 0.01, resp.). A comparison of the effects of free GMDP and the GMDP, immobilized on the AMNPs, showed that the effect of the latter exceeded the effect of free drug on the expression of CD36 molecules (*P* < 0.05). Surprisingly, we did not find the significant changes in the expression of the scavenger receptors by macrophages after its incubation with GMDP, immobilized on the UMNPs, in comparison to the control values (*P* > 0.05 in all cases). A comparative analysis of the effects of different nanocomposites demonstrated that the amount of macrophages with the surface expression of CD36 molecules after stimulation by GMDP, immobilized on the UMNPs, was significantly lower than analogous result received for GMDP immobilized on the AMNPs (*P* < 0.01). Evidently, the immobilization of GMDP on the UMNPs leads to loss of the immunomodulatory effect of the drug. The same effect was observed when we studied the influence of the free and the immobilized GMDP upon the synthesis of NOD2 and RAGE molecules by macrophages (Figure 4).

We found that after 24 h incubation of macrophages with free GMDP the insignificant increase of NOD2 mRNA expression by macrophages was observed comparing to the control values. After immobilization GMDP onto the AMNPs, the significant increase both of NOD2 and RAGE mRNAs expression by peritoneal macrophages of women with endometriosis was seen (*P* < 0.05 in both cases). We did not find significant differences in the expression of NOD2 and RAGE mRNAs by macrophages incubated with GMDP, immobilized upon UMNPs. In this case, similar to the experiments with scavenger receptors, the immobilization of GMDP upon UMNPs led to the decrease of the immunostimulatory action of the drug. Possibly unlike AMNPs the adsorption interaction of GMDP with surface functional groups of UMNPs results in conformational changes of the drug molecules. This leads to the loss of the immunomodulatory effect of GMDP.

We also studied the action of GMDP upon synthesis of SOCS1 by peritoneal macrophages (Figure 4(c)). This suppressor factor plays the important role in the regulation of the macrophages activation via PRRs molecules, preventing the undesirable hyperactivation of phagocytes [34]. Earlier it was shown that MPD and its derivatives downregulate the expression of SOCS1 [35]. In our work we also observed the significant diminishment of SOCS1 mRNA expression by peritoneal macrophages after its incubation in the presence of free GMDP or GMDP, immobilized onto the UMNPs (Figure 4(c)) and these results are in a good accordance to the literature data. But after incubation of peritoneal macrophages with GMDP, immobilized upon AMNPs, the level of SOCS1 mRNA expression did not differ from that of unstimulated macrophages. Likely, this phenomenon might be connected with the significant stimulatory action of GMDP, immobilized onto the AMNPs. To restore the balance between the pro- and anti-inflammatory signals, entering in the macrophages after its stimulation by GMDP, the high level of suppressor activity is kept in the cells. It has been shown that macrophages produce a huge amount of different biologically active molecules, which are involved in the realization of different stages of phagocytosis [36]. In our study we estimated the effect of GMDP on the synthesis of proteolytic enzymes relevant to the family of matrix metalloproteinases (MMPs). It is known that MMPs are proteolytic enzymes involved in extracellular matrix and basement membranes degradation. MMPs activity is negatively regulated by its specific inhibitors - TIMPs (tissue inhibitors of matrix metalloproteinases) [37]. It has been shown that MMPs play an active role in the development of many pathological conditions, including tissue destruction, cancer invasion and metastasis, angiogenesis, and apoptosis [37]. Supposedly, MMPs may be actively involved in pathogenic mechanisms of endometriosis development. Recent studies have demonstrated that the content of MMP-1, MMP-2, and MMP-7 in the peritoneal fluid of women, with endometriosis is increased in comparison to that of healthy women, and the level of their inhibitors (TIMPs), on the contrary, is reduced [38]. Our earlier investigations have showed the imbalance of MMPs and their inhibitors synthesis in the endometrial tissue of women with endometriosis as well as during the development of experimental endometriosis in rats [39]. However, according to the literature data, production of MMP-9 by macrophages of women with endometriosis is suppressed. Expression and secretion of matrix metalloproteinase MMP-9 by the macrophages serve to degrade the extracellular matrix of cells that are designated for phagocytosis [40]. So, the decrease of MMP-9 expression may be the cause of the impairment of phagocytotic capability of the peritoneal macrophage of patients with endometriosis.

**Figure 5:**
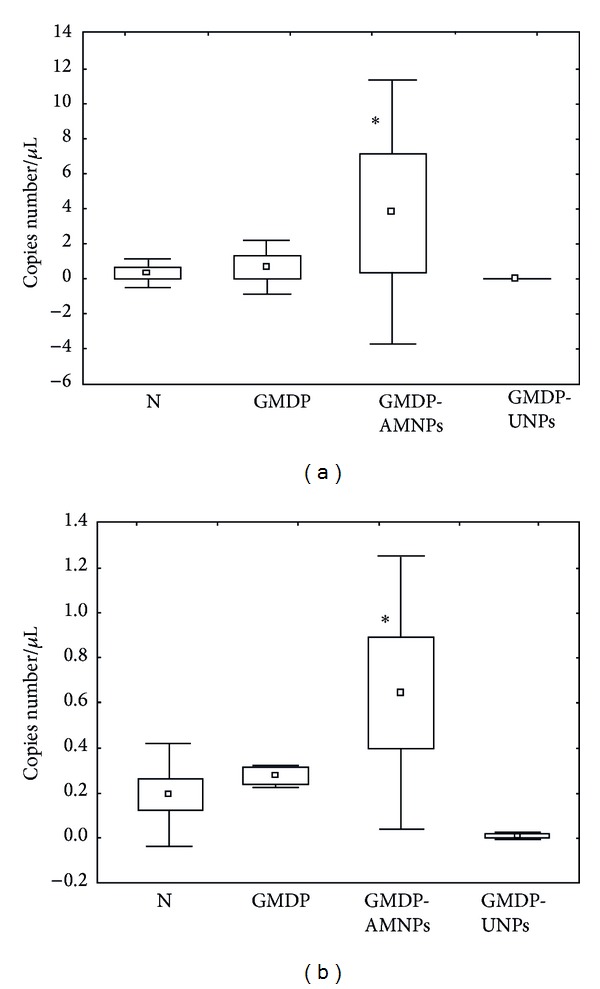
The influence of free GMPD and GMPD, immobilized on different silica nanoparticles, upon the MMP-9 and TIMP-1 mRNAs expression by peritoneal macrophages of women with endometriosis. (a) The influence of GMPD on MMP-9 mRNA expression. (b) The influence of GMDP on TIMP-1 mRNA expression. (Notes: C—control (incubation in RPMI 1640 medium only), GMDP—free form of GMDP, AMNPs-GMDP, immobilized onto the AMNPs, UMNPs-GMDP, immobilized onto the UMNPs, results are presented as the mean ± standard error; *differences in comparison to the control values are statistically significant, *P* < 0.05).

Our experiments in vitro showed that after 24-hour incubation of the peritoneal macrophages with free GMDP the level of MMP-9 mRNAs expression slightly increased in comparison to the control values (*P* > 0.05) (Figure 5(a)).

No significant effect of free GMDP on the expression of TIMP-1 was observed. Incubation of the peritoneal macrophages with GMDP immobilized on AMNPs resulted in significantly increase of the MMP-9 mRNA expression in comparison to that for the unstimulated macrophages (Figure 5(a)). It should be noted that in this case the simultaneous increase of the TIMP-1 mRNA expression was seen. It is known that a high level of MMPs expression can lead to inadequate activation of the macrophages [37]. Therefore, the simultaneous increase of MMP-9 and its inhibitor synthesis can be considered as a positive phenomenon, preventing the unbalanced macrophages activation. Thus, we observed the same inducing action of GMDP, immobilized onto the AMNPs, upon the synthesis of factors, activating macrophages, and its specific inhibitors without prevalence of activatory signals.

On the contrary, incubation of the macrophages with GMDP, immobilized on UNPs, led to the decrease of TIMP-1 mRNA expression by the macrophages (*P* < 0.05). The expression of MMP-9 mRNA in this case was lower than the detectable values (Figure 5).

## 4. Conclusions

The obtained results have demonstrated the possibility of application of hybrid silica nanomaterials for topical delivery of GMDP to the peritoneal macrophages. Our studies in vitro have showed that the immunomodulatory effect of GMDP can be intensified by the immobilization of GMDP on silica nanoparticles. Two types of the silica nanoparticles have been investigated as nanocarriers for the drug: unmodified silica nanoparticles (UMNPs) and modified by aminopropyl groups silica nanoparticles (AMNPs). In vitro studies have showed that although UMNPs, prepared by sol gel synthesis using sugar as template, exhibit a high ability to interact with the peritoneal macrophages, they cannot be applied as nanocarriers for topical delivery of GMDP to the peritoneal macrophages of women with endometriosis. The immobilization of GMDP on the nanoparticles of this type led to the complete suppression of the stimulatory effect of GMDP on the expression of PRRs molecules and synthesis of proteolytic enzymes from the matrix metalloproteinases (MMPs) family. The topical delivery of GMDP to the peritoneal macrophages by AMNPs led to the enhance the initially reduced membrane expression of SRs by the macrophages of women with endometriosis, increased NOD2 and RAGE mRNAs expression, and also promoted an increase of the expression of MMP-9 mRNA. Simultaneously elevated synthesis of factors, preventing undesirable overactivation of peritoneal macrophages (SOCS1 and TIMP-1) was observed after the action of GMDP, immobilized onto AMNPs. The effect of the AMNPs immobilized GMDP in some cases exceeded the effect of free GMDP. Thus, comparison of two types of silica nanoparticles as possible nanocarriers for topical delivery of immunomodulatory drug GMDP in peritoneal macrophages has demonstrated that AMNPs are the most suitable nanocarrier for the drug.

## Figures and Tables

**Table 1:** The influence of 24-hour incubation of peritoneal macrophages with different types of silica nanoparticles upon functional activity of macrophages.

Parameters, %	Control (RPMI 1640 medium) (*n* = 10)	AMNPs (*n* = 5)	UMNPs (*n* = 8)
HLA-DR+	80.98 ± 2.76	85.90 ± 1.58	79.53 ± 4.39
CD11b+	80.65 ± 2.53	82.10 ± 1.67	80.37 ± 4.80
CD95+	55.48 ± 2.27	54.45 ± 4.32	60.27 ± 4.45
CD36+	63.11 ± 4.01	64.50 ± 3.39	67.63 ± 6.54
CD204+	58.11 ± 3.62	55.34 ± 2.26	61.00 ± 3.32
NBT sp	17.00 ± 1.60	10.00 ± 5.00	12.80 ± 2.03
NBT st	24.70 ± 1.62	18.50 ± 5.50	22.80 ± 2.11

**Table 2:** Characteristics of the intensity of interaction between peritoneal macrophages and different silica nanoparticles after 1-hour and 24-hour incubation.

Incubation time	AMNPs (*n* = 9)	UMNPs (*n* = 10)
1 hour	75.31 ± 2.91	82.90 ± 2.50
24 hours	80.23 ± 2.77	94.40 ± 1.95
	*P*_1_ > 0.05	*P*_1_ < 0.05
		*P*_2_ < 0.01

*P*: The level of statistically significant differences between different time of incubation and between different types of silica nanoparticles, *P*_1_ is given in relation to the data after 1-hour incubation; *P*_2_ is given in relation to the data for AMNPs.

**Table 3:** Comparative characteristics in vitro of effects of free GMDP and GMDP immobilized on UMNPs and AMNPs on spontaneous expression of functional and SR molecules by peritoneal macrophages of women with endometriosis.

Parameter, %	Macrophages + RPMI 1640 medium (control) (*n* = 31)	Macrophages + free GMDP (*n* = 16)	Macrophages + GMDP immobilized on AMNPs (*n* = 7)	Macrophages + GMDP immobilized on UMNPs (*n* = 6)
CD36 + (SR-B)	63.73 ± 2.75	75.67 ± 3.15**	84.10 ± 1.55^∗∗∗x^	65.78 ± 4.23^yy^
CD204 + (SR-AI)	55.11 ± 2.18	69.79 ± 3.75***	69.48 ± 3.64**	59.73 ± 4.47

*Statistically significant differences between data received for stimulated macrophages and control values (**P < 0.01, ***P < 0.01).

^x^Statistically significant differences between data received for free GMDP and GMDP, immobilized on AMNPs (^x^ *P* < 0.05).

^y^Statistically significant differences between data received for GMDP, immobilized on AMNPs and UMNPs (^yy^ *P* < 0.01).
